# The Swansea Hospital

**Published:** 1897-10-23

**Authors:** 


### THE SWANSEA HOSPITAL.

Among the various matters which came before the last monthly meeting of the board of management of the Swansea Hospital was the resignation of the chairman of the House Committee. On the matters at issue we do not wish to say anything. Differences will sometimes occur within a committee, and although in theory a chairman can but act as the mouthpiece of those over whom he presides, we know so well how immensely important it is to such an institution as a hospital to have as its chairman someone who is actively interested in its success and in the various details of its management, that we can well excuse such an official becoming a little autocratic in his methods, and we cannot but express our regret that Colonel Morgan should have felt called upon to resign a post which he has occupied for so long. It seems to us, however, a great pity that an air of mystery should have been thrown over the proceedings of the meeting by the exclusion of reporters. Neither the late chairman nor the House Committee can well leave the matter as it is. It cannot be fair either to the subscribers or to the public to allow the notion to get abroad that there is something seriously wrong in the affairs of the hospital if the difference is only a trivial or personal matter; while, if it touches any vital question, the subscribers ought to know all about it. In such matters the truth always has in the end to come out, and it is better that the public should be at once placed in possession of the facts than that it should be left to its own imaginings to the detriment of the institution.

